# Sterile Vegetations in Malignancy: A Rare Case of Nonbacterial Thrombotic Endocarditis in a Patient with Metastatic Melanoma

**DOI:** 10.3390/reports9020129

**Published:** 2026-04-22

**Authors:** Libardo Rueda Prada, Alejandro Fabrega Gerbaud, Marta Berguido de la Guardia, Juan C. Martinez Morales, Carlos A. Velandia-Carrillo, Carlos Vergara Sanchez

**Affiliations:** 1 Division of Hospital Internal Medicine, Mayo Clinic, Jacksonville, FL 32224, USA; 2 Acute Care Research Consortium, Mayo Clinic, Jacksonville, FL 32224, USA; 3 Faculty of Medicine, Universidad Autonoma de Bucaramanga, Santander 681004, Colombia; 4 Department of Cardiology, Fundacion Clinica Foscal Internacional, Santander 681004, Colombia; 5 Department of Cardiology, Mayo Clinic, Jacksonville, FL 32224, USA

**Keywords:** endocarditis, non-infective, melanoma, thrombosis, hypercoagulability, paraneoplastic syndromes

## Abstract

**Background and Clinical Significance:** Nonbacterial thrombotic endocarditis (NBTE) is a sterile fibrin-platelet valvular condition associated with malignancy and hypercoagulable states. It produces friable vegetations prone to systemic embolization, often presenting as multifocal ischemic stroke. While modestly linked to advanced adenocarcinomas, its association with melanoma is exceedingly rare; **Case Presentation:** We present a 43-year-old man with recently diagnosed metastatic melanoma who presented with fever, confusion and abdominal pain. Brain magnetic resonance imaging (MRI) revealed multifocal bilateral acute infarcts. Additional imaging demonstrated splenic and bilateral renal infarcts. Transesophageal echocardiography (TEE) revealed an 8 mm × 7 mm multilobar lesion on the posterior mitral valve leaflet. Blood cultures remained persistently negative; autoimmune and infectious workup were unrevealing, and positron emission tomography-computed tomography (PET-CT) showed no cardiac hypermetabolism. Despite empiric antibiotics for suspected infective endocarditis (IE), progressive embolic infarcts occurred. After exclusion of infection, NBTE was considered, and therapeutic enoxaparin was initiated, resulting in clinical stabilization without hemorrhagic conversion; **Conclusions:** Distinguishing NBTE from IE remains challenging due to overlapping and nonspecific imaging findings. TEE is the preferred diagnostic modality because of its high sensitivity for detecting small valvular vegetations. Adjunctive imaging modalities such as brain MRI and PET-CT may support the diagnosis by demonstrating embolic patterns or excluding metabolically active infectious vegetations. Management primarily relies on systemic anticoagulation, while percutaneous vegetation aspiration may represent a potential diagnostic and therapeutic strategy. Clinicians should maintain high suspicion of this condition in patients with advanced melanoma and other malignancies presenting with multifocal embolic phenomena and negative cultures to enable timely anticoagulation.

## 1. Background

Nonbacterial thrombotic endocarditis (NBTE), also known as marantic endocarditis, is a form of non-infective endocarditis characterized by sterile valvular vegetations composed primarily of fibrin and platelets in the absence of microorganisms [1]. NBTE has been described as occurring in hypercoagulable states such as systemic lupus erythematosus, antiphospholipid antibody syndrome, burns and sepsis, and is most strongly associated with malignancy [1]. Pathologically, NBTE vegetations are friable, lack significant inflammatory reaction, and are typically located along the edge of valve leaflets, most often affecting previously healthy mitral and aortic valves [1,2,3]. Their fragile structure predisposes to fragmentation and systemic arterial embolization, frequently resulting in stroke and visceral organ infarction [1]. Notably, approximately 50% of cancer-associated embolic strokes are classified as embolic strokes of undetermined source and most involve multiple vascular territories [4], making NBTE especially relevant among this patient population.

Evidence regarding NBTE in cancer patients derives largely from case reports and autopsy studies. Autopsy data suggest that NBTE may be underrecognized, with reported prevalence ranging from 0.93% to 1.08% in autopsy series of the general population [3,5]. In two large studies, 32–59% of NBTE cases were associated to cancer, but endocarditis had not been considered as a potential embolic source prior to death [3,5]. NBTE suspicion tends to arise only after embolic vascular events, and diagnosis is typically established through echocardiographic evaluation [1]. Transthoracic echocardiography (TTE) is commonly used as an initial screening modality for valvular vegetations and may be followed by the more sensitive transesophageal echocardiography (TEE) when TTE findings are negative or inconclusive [1,6]. In a prospective study of patients with solid tumors, valvular vegetations were detected by TTE in 19% of cases, and 24% of those patients experienced thromboembolic complications [2]. Meanwhile, TEE has a reported sensitivity and specificity of 87–100% and 91–100%, respectively, for IE diagnosis in patients with native valves [7].

The association of NBTE with malignancy is well established but has been historically reported and studied in patients with hematologic malignancies and solid tumors primarily of the lung, urinary tract and reproductive organs as well as malignancies of the GI tract like stomach and pancreatic cancer [2,3,6,8]. The incidence of NBTE in patients with metastatic melanoma or primary cutaneous malignancies remains unknown due to its infrequent association. The published literature regarding NBTE in patients with melanoma is limited to one 1994 French case report and an autopsy-based study from 1987 in which melanoma was identified in 3 of 116 patients with NBTE-associated cerebral infarction [8,9]. Due to the rarity of this association among the recent literature, we present the case of a 43-year-old man with metastatic melanoma who was found to have NBTE. This case highlights a devastating manifestation of malignancy-associated hypercoagulability and reinforces the importance of considering NBTE in cancer patients presenting with unexplained systemic embolic phenomena.

## 2. Case Description

**Figure 1:**
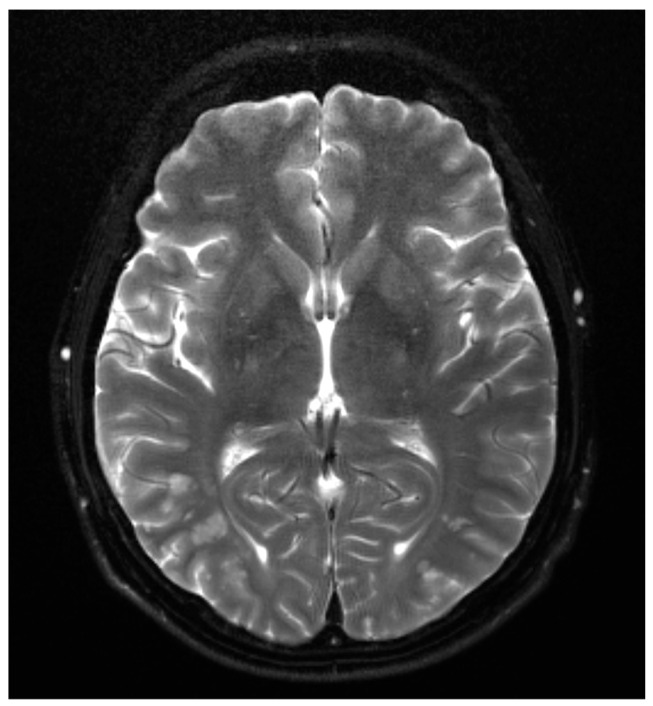
Brain MRI: Brain MRI showing numerous small bilateral cerebral and cerebellar infarcts predominantly involving watershed territories.

A 43-year-old man with a past medical history of BRAF mutated melanoma in his left posterior upper arm—Breslow depth 0.8 mm, status post wide local excision with negative sentinel lymph node biopsy in 2019—and recent liver biopsy 4 days before this presentation which was diagnostic of metastatic melanoma, presented to the emergency department complaining of fever, chills, intermittent confusion, shortness of breath, and diffuse abdominal discomfort for the past 2–3 days. A brain magnetic resonance imaging (MRI) completed earlier on the day of presentation was concerning for numerous small bilateral acute to early subacute cerebral and cerebellar infarcts. These predominantly involved the watershed territories, had no associated mass effect or hemorrhage, were likely cardioembolic in nature, and presented multiple small areas of leptomeningeal enhancement (Figure 1).

On presentation, vital signs showed tachycardia with a heart rate of 116 bpm, temperature 36.7 °C, blood pressure 113/79 mmHg, respiratory rate 17 rpm, and no hypoxia. The patient was alert and in no acute distress. Physical examination revealed no conjunctival or mucosal lesions and no palpable lymphadenopathy. Cardiac examination demonstrated normal S1 and S2 with tachycardia but no murmurs or rubs. Lung auscultation was clear without rales or wheezing and there were no signs of respiratory distress. The abdomen was soft with mild diffuse tenderness to palpation but without guarding or rebound. Neurological examination showed no focal deficits. Skin examination revealed no classic stigmata of endocarditis except for splinter hemorrhages in the fingernails of both hands.

**Figure 2:**
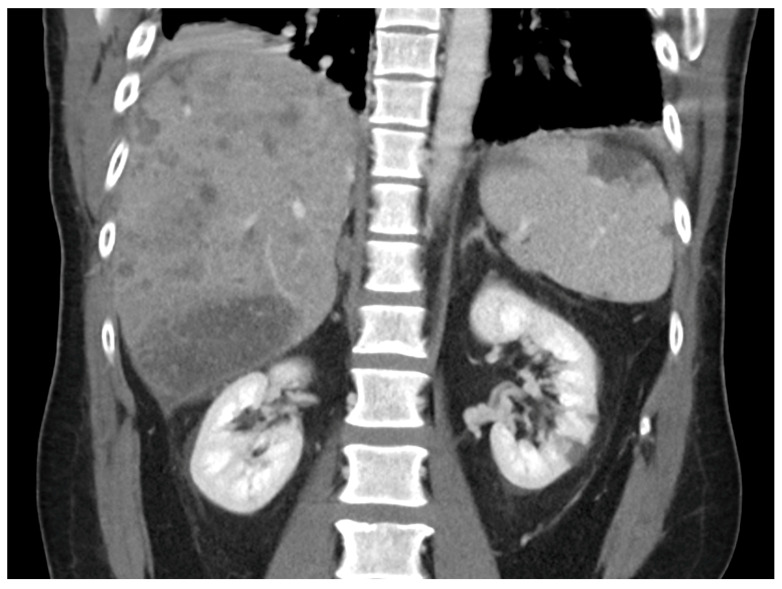
Abdominal CT: Abdominal CT with IV contrast showing acute splenic and bilateral renal infarcts suggestive of an embolic phenomenon. Diffuse hepatic metastasis can also be observed.

**Figure 3:**
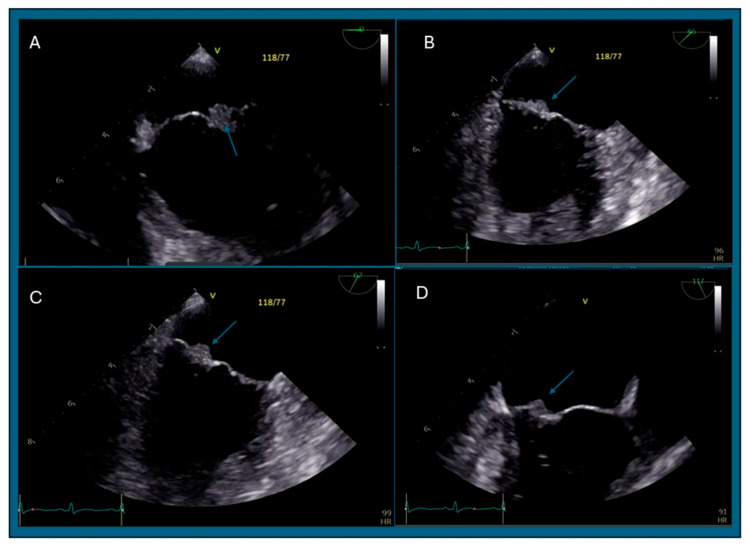
TEE of marantic endocarditis: TEE views of the mitral valve at (**A**) 0 degrees (**B**) 46 degrees (**C**) 62 degrees and (**D**) 117 degrees where a complex multilobular, immobile echodensity (blue arrow) is seen at the atrial side of the posterior leaflet of the mitral valve measuring 8 mm × 7 mm.

Initial workup was remarkable for leukocytosis 11.4 × 10^9^/L, hemoglobin 14.8 g/dL, acute thrombocytopenia 109 × 10^9^/L, lactic acid 2.1 mmol/L, unremarkable chemistry panel except for transaminitis with aspartate aminotransferase (AST) 398 U/L, alanine aminotransferase (ALT) 274 U/L, alkaline phosphatase 274 U/L, total bilirubin 1.8 mg/dL (direct bilirubin 1.2 mg/dL), albumin 3.4 g/dL, INR 1.5 and blood cultures were collected. Electrocardiogram (ECG) showed sinus tachycardia and no ischemic changes. A chest computed tomography (CT) angiogram showed no consolidation, and no acute pulmonary embolism. A CT abdomen and pelvis with intravenous (IV) contrast revealed acute splenic and bilateral renal infarcts suggestive of an embolic phenomenon, diffuse hepatic metastasis, no acute complication related to recent liver biopsy, and diffuse osseous metastatic disease (Figure 2). Empiric antibiotic treatment was initiated with IV vancomycin and aztreonam (due to history of severe penicillin allergy) to cover possible infective endocarditis (IE) given concerns for bacterial translocation from recent liver biopsy. Neurology recommended against anticoagulation until IE was ruled out. A CT angiogram of the head and neck showed patent intracranial and extracranial vessels without identifiable embolic source. A TEE revealed a complex, multilobular, immobile echo density on the posterior mitral valve leaflet measuring 8 mm × 7 mm with minimal extension onto the anterior leaflet suggestive of endocarditis vs. thrombus vs. metastasis (Figure 3). These findings and the absence of valvular destruction, abscess formation, significant regurgitation or shunts, further favored a noninfectious etiology, like NBTE. A ceftriaxone test dose was completed with no side effects, so aztreonam was switched to IV ceftriaxone on day 2. Antinuclear antibodies, antiphospholipid antibodies, beta 2 microglobulin, lupus anticoagulant, and cryoglobulins were negative. HIV Ag/Ab screen was negative and RPR non-reactive.

**Figure 4:**
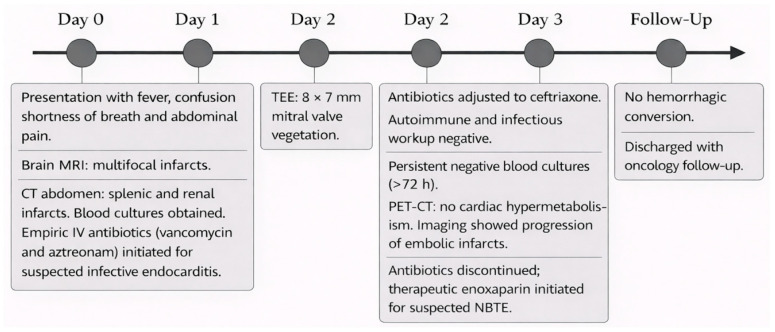
Clinical timeline.

The patient had no new episodes of fever; however, three days after admission he developed worsening epigastric abdominal pain. Repeat CT of the abdomen and pelvis showed worsening infarcts in the splenic and bilateral renal artery territories as well as new parietal infarcts on brain imaging. Initial blood cultures remained negative for more than 72 h. A positron emission tomography-computed tomography (PET-CT) scan showed extensive hepatic and osseous metastatic disease, but no focal cardiac hypermetabolism. Risk of brain acute infarct hemorrhagic conversion was considered low, based on small size of stroke and lack of susceptibility weighted imaging change. After 3 days of empiric IV antibiotics, which were discontinued following persistently negative blood cultures and further evaluation supporting a noninfectious etiology, the patient was initiated on therapeutic enoxaparin at 1 mg/kg twice daily for suspected marantic endocarditis. Non-contrast head CT at 24 h did not show any signs of hemorrhagic conversion. MRI of the spine did not show evidence of leptomeningeal disease. The patient was scheduled to follow up with Oncology after hospital discharge with a plan to start immunotherapy. A Karius test collected during admission came back negative days after the patient was discharged from the hospital (Figure 4).

## 3. Discussion

Cancer is a leading cause of NBTE, most frequently associated with advanced mucin-producing adenocarcinomas particularly of the pancreas, lung, stomach, ovary, and biliary tract [1]. Literature reporting NBTE from melanoma is very scarce. A 1994 report by Bouloc et al. described NBTE complicating metastatic melanoma with multifocal ischemic strokes and vegetations visualized in echocardiography [9]. The earlier literature includes isolated reports of paraneoplastic thrombotic phenomena in melanoma, including eosinophilic parietal thromboendocarditis [10], but true valvular NBTE associated with melanoma remains exceptionally uncommon. Compared with the more typical pancreatic or lung adenocarcinoma NBTEs [11,12], our case is notable for several reasons. Melanoma is a non-mucinous malignancy and is not classically associated with NBTE. In addition, the patient presented with simultaneous multi-organ embolization (brain, spleen, kidneys) and the diagnosis of NBTE occurred early in the metastatic course, only four days after confirmation of hepatic metastases. Similarly to the case described by Bouloc et al. [9], our patient presented with multifocal cerebral infarcts suggestive of cardioembolism; however, he additionally developed progressive systemic embolization before anticoagulation could be initiated, highlighting the fulminant thrombotic potential of this condition.

Differentiating IE from NBTE remains a major clinical challenge. NBTE vegetations lack inflammatory destruction and are often small, friable, and emboligenic [1]. Unlike IE, fever may be absent or low-grade, murmurs are often subtle, and blood cultures remain persistently negative [13]. In our case, the presence of splinter hemorrhages and fever initially raised concern for IE, appropriately prompting broad-spectrum antibiotics. However, persistently negative blood cultures beyond 72 h and negative autoimmune testing shifted suspicion toward malignancy-related NBTE. This diagnostic evolution mirrors reports where NBTE masqueraded as culture-negative endocarditis. TTE can detect the large, mobile and irregular vegetations that characterize IE, as well as the valve destruction process and regurgitation that may accompany it. Whereas TTE frequently misses small vegetations, TEE has higher sensitivity for lesions <5 mm [13]. For instance, our patient’s TEE revealed vegetation morphology consistent with a non-infective etiology. Adjunctive imaging modalities may aid differentiation, such as MRI or PET-CT. Brain MRI demonstrated multiple bilateral infarcts across several vascular territories, consistent with an embolic shower pattern. Although described in NBTE, this finding is not specific and may also occur in IE, where septic emboli can additionally manifest with microabscesses or hemorrhagic lesions [14]. PET-CT may also help to exclude metabolically active infectious vegetations [15]. However, this is an expensive and adjunct, not a “stand-alone” diagnostic modality. In our case, the absence of focal cardiac hypermetabolism supported a sterile process.

In challenging and diagnostically ambiguous cases, percutaneous vegetation aspiration has been described both for diagnostic confirmation and to reduce embolic burden [16]. Although more commonly utilized in right-sided or large vegetations, aspiration allows histopathologic evaluation to distinguish sterile thrombus from infective vegetations or metastatic tumor deposits [17]. Recent work describes successful utilization for aspiration on left-sided cardiac chamber endocarditic vegetations [18]. Other reports highlight its procedural success in cases of NBTE, where it can safely reduce vegetation size, especially for high-risk surgical candidates [16]. Given that NBTE vegetations are friable and highly embolic, aspiration may theoretically reduce embolic load in selected patients with ongoing embolization despite anticoagulation [19]. In our patient, the lesion was immobile, relatively small, and responded well to systemic anticoagulation; therefore, invasive removal was not pursued. This strategy may merit consideration in future melanoma-associated NBTE cases with refractory embolization.

NBTE represents an extreme manifestation of malignancy-driven hypercoagulability. Tumor cells interact with monocytes and endothelial cells, promoting tissue factor expression and thrombin generation, leading to platelet–fibrin deposition on previously normal valves [1]. Disseminated intravascular coagulation (DIC)-like features, including thrombocytopenia and elevated INR, are frequently observed [20]. In fact, laboratory evidence of DIC occurs in approximately 10–15% of patients with metastatic cancer [21] while histologic features of DIC are present in around 50% of patients with NBTE, often in association with hyperfibrinogenemia and thrombocytopenia [13]. Our patient’s acute thrombocytopenia and elevated INR may reflect this prothrombotic–consumptive state elevating embolic risk, reflected by his progression of splenic and renal infarcts before anticoagulation was initiated. Of all, cerebrovascular disease is the most common clinical manifestation and often the initial presentation of the underlying disease [13]. Notably, NBTE accounts for approximately one-quarter of all ischemic strokes in patients with malignancy [22].

Anticoagulation with unfractionated heparin or low molecular weight heparin (LMWH) is the cornerstone of therapy [1,13], with LMWH often favored in cases of malignancy-associated thrombosis [23]. Unlike IE, where anticoagulation may increase the risk of intracranial hemorrhage, NBTE does not involve infectious arterial wall destruction, making anticoagulation comparatively safer once hemorrhagic transformation risk is determined [23,24]. Neurologists assess hemorrhagic conversion risk using infarct size, imaging features (absence of susceptibility-weighted hemorrhage), timing since stroke, and clinical stability [25]. In our patient, the small infarct size, absence of hemorrhagic signal on imaging, and stable neurologic examination allowed initiation of therapeutic enoxaparin. Follow-up CT confirmed no hemorrhagic conversion.

## 4. Conclusions

NBTE remains a rare but devastating complication of malignancy that demands heightened clinical awareness. Although classically associated with advanced adenocarcinomas, this case underscores that metastatic melanoma can also predispose to NBTE and catastrophic systemic embolization. For clinicians, early recognition is critical. In patients with active cancer who present with multifocal ischemic strokes, negative blood cultures, and echocardiographic evidence of valvular vegetations, NBTE should be strongly considered. Prompt differentiation from IE is essential, as management strategies differ substantially and delays in appropriate anticoagulation may result in recurrent embolic events. Given the high morbidity and mortality associated with cancer-related NBTE, maintaining a high index of suspicion is critical to allow timely diagnosis, prompt anticoagulation, and appropriate oncologic management in this high-risk population.

## Data Availability

The original contributions presented in this study are included in the article. Further inquiries can be directed to the corresponding author.

## Abbreviations

The following abbreviations are used in this manuscript:

NBTE	Non Bacterial Thrombotic Endocarditis
TTE	Trans Thoracic Echocardiography
TEE	Trans Esophageal Echocardiography
MRI	Magnetic Resonance Imaging
CT	Computed Tomography
IE	Infective Endocarditis
PET-CT	Positron Emission Tomography-computed tomography
DIC	Disseminated Intravascular Coagulation
LMWH	Low Molecular Weight Heparin
IV	Intravenous
